# Actigraphy and subjective sleep predictors of nine-year generalized anxiety disorder

**DOI:** 10.1016/j.janxdis.2025.103095

**Published:** 2025-11-19

**Authors:** Nur Hani Zainal, Natalia Van Doren

**Affiliations:** a National University of Singapore, Department of Psychology, Kent Ridge Campus, Singapore; b University of California at San Francisco, Department of Psychiatry and Behavioral Sciences, San Francisco, CA, USA

**Keywords:** Actigraphy, Circadian sleep-wake rhythm, Generalized anxiety disorder, Insomnia, Longitudinal, Worry

## Abstract

**Background:**

Sleep disturbances have been linked to generalized anxiety disorder (GAD) symptoms. However, cross-sectional studies, linearity assumptions, and limited predictor sets preclude identifying which unique sleep disturbance markers precede GAD symptoms. We thus harnessed machine learning (ML) to determine objective and subjective sleep disturbance predictors of nine-year GAD symptoms.

**Methods:**

Community adults (*N* = 1054) underwent baseline surveys, clinical interviews, and seven-day sleep actigraphy protocols. GAD symptoms were reassessed nine years later. Seven ML models were examined with 44 baseline predictors. Partial dependence and Shapley additive explanation plots were created as interpretable ML approaches with the best-performing random forest model using nested cross-validation. Sensitivity analyses included and excluded GAD sleep items.

**Results:**

The final multivariable predictive algorithm performed well (*R*^*2*^ = 69.7 %, 95 % confidence interval [67.3 %–71.9 %]), thus explaining over half the variance in the outcome. These self-reported sleep disturbances predicted GAD symptoms in descending order of relative importance: sleep disturbances, poorer sleep quality, longer sleep onset latency, daytime dysfunction, habitual sleep inefficiency, and sleep medication use. These rest-phase actigraphy markers predicted nine-year GAD symptoms: higher maximum and total activity counts. Longer total sleep time during the sleep phase and higher average sleep bouts during the active phase also predicted nine-year GAD severity.

**Conclusions:**

Outcomes highlight the importance of combining actigraphy and self-report sleep assessments. Future studies should determine the degree to which these patterns extend to the within-person level to develop early prevention, treatment, and precision mental health strategies for individuals at risk of, or with, increased GAD severity.

## 1. Introduction

Generalized anxiety disorder (GAD) is a common mental disorder marked by excessive and uncontrollable worry linked to myriad symptoms, such as concentration issues, irritability, and muscle tension, over at least the past six months (American Psychiatric Association, 2022). Epidemiological studies have indicated that the global lifetime prevalence rates of GAD range from 1.6 % to 5.0 % across 27 countries with varying income levels (Ruscio et al., 2017; Watterson et al., 2017). Persistent and severe GAD symptoms have been associated with worse mental health comorbidities, physical health, and psychosocial functioning (Patriquin & Mathew, 2017; Shin & Newman, 2019). Thus, determining the distal risk factors (i.e., long-term preceding variables) of GAD is crucial for developing optimal prevention and treatment approaches.

Sleep disturbances might be critical distal risk factors of pathological worry and related GAD symptoms. However, the unique pathways via which sleep disturbances predict persistent worry remain understudied. One plausible mechanism involves dysregulations in daily activity-rest-sleep cyclical routines, which actigraphy wearables could capture seamlessly while complementing self-reports of sleep quality. Homeostatic sleep disturbances, such as longer self-reported sleep onset latency (SOL) and higher total sleep time (TST), might forecast long-term increased GAD by maintaining hyperarousal symptoms (Kalmbach et al., 2018). Simultaneously, reduced activity counts during active wake phases and rest or sleep fragmentation during delimited rest or sleep phases might signal circadian disarray. Such sleep disturbances are germane to well-established frameworks of anxiety disorders, since decreased diurnal physical activity and erratic rest and sleep phases have been associated with cognitive dysfunction (Kong et al., 2023), emotion dysregulation (Palmer et al., 2024), increased arousal (Baker et al., 2017), and repetitive negative thinking (Clancy et al., 2020). Although actigraphy wearables do not directly assess endogenous circadian stages (e.g., melatonin levels), they can indicate circadian-governed routine behaviors through activity and sleep patterns during active wake, rest, and sleep phases. Together, combining self-report and actigraphy indices of daily activity, rest, and sleep routines could help identify unique markers of sleep disturbance predictive of long-term GAD severity.

Although existing data partly support these theories, most evidence has been cross-sectional, which limits directional inference. A meta-analysis of 55 studies found a consistent negative association between worry and sleep quality, but the cross-sectional focus of research on SOL, TST (Clancy et al., 2020), and related indices (Konjarski et al., 2018) precludes causal conclusions (Rubin, 2008). Experience-sampling studies show that sleep disturbances often (Thielsch et al., 2015; Zhou et al., 2024b), although not always (McGowan et al., 2016), precede higher worry; however, most span only days, limiting insight into long-term effects. Reviews suggest that actigraphy-based measures reveal more consistent bidirectionality between sleep and affective disturbances across populations (Hickman et al., 2024; Ten Brink et al., 2022). Poorer sleep quality also predicts future worry across months and years among adolescents (Richardson et al., 2024; Xiao et al., 2023), adults (Nguyen et al., 2022), and student nurses (Xu et al., 2020). Although self-reports remain useful, they overlook cyclical activity-rest-sleep indices captured by actigraphy. Overall, sleep disturbances across metrics plausibly confer distal risk for GAD symptoms.

Examining both self-reported and actigraphy sleep disturbances as predictors of long-term GAD symptoms is essential for myriad reasons. This holistic method captures complex relations between objective and subjective indices of sleep disturbances. Subjective self-reports often emphasize individual appraisals of fatigue, insomnia, and restlessness, which are frequently observed in GAD; however, objective actigraphy wearables may not correlate with these perspectives (Melo et al., 2021). The potential lack of correlation between subjective restlessness and objective movement, as assessed by actigraphy wearables, has been termed the “reactivity paradox” (Pastre & Lopez-Castroman, 2022). Thus, while subjective (self-report) measures of sleep disturbance can provide insights into an individual’s perceptions and experience of sleep difficulties, actigraphy wearables can offer more objective information on sleep-wake circadian patterns and rhythms, which might be compromised in those at risk of future GAD symptoms. These patterns may be more subtle and thus may not always enter a person’s conscious awareness, yet may plausibly impact risk for GAD. For instance, actigraphy could detect decreased sleep-wake rhythms and reduced physical activity that might not be evident from self-reports (Difrancesco et al., 2019; Franklin et al., 2021). Overall, integrating objective and subjective measures enhances the ability to study the prognostic value of sleep disturbances in predicting long-term GAD symptoms.

On that note, using both actigraphy and self-report measures of sleep disturbances to predict long-term GAD symptoms is crucial for comprehensively understanding sleep-related risk factors for GAD. This approach considers the impact of both subjective perceptions and more objective biological metrics. Such data inevitably results in a high-dimensional predictor set. Traditional ordinary least squares regression is ill-suited to manage collinearities among variables in a high-dimensional predictor set (Tomaschek et al., 2018). Machine learning (ML) is a data-driven technique within the suite of precision medicine methods and is a valuable alternative to circumvent these issues by effectively handling multicollinearity (Rosenbusch et al., 2021). Moreover, other benefits of using ML rather than ordinary least squares regression to examine how sleep disturbances predict future GAD symptoms include its ability to model higher-order interactions and complex nonlinear associations (Sajno et al., 2022). These ML strengths are key, as research has shown that the relations between sleep disturbances and worry symptoms could be nonlinear and dependent on other variables (McWood et al., 2022; Shimizu et al., 2020). Furthermore, ML enables clinicians and researchers to determine whether an observed pattern generalizes to novel data using nested cross-validation, which separates training and test data subsets (Zhou et al., 2024a). To this end, such prognostication efforts can better inform prevention and treatment approaches, indicating whether an actionable ‘prognostic calculator’ (Oliver et al., 2021) can be built to predict long-term GAD symptoms if such patterns are replicated in external validation studies (Collins et al., 2024).

Objective actigraphy and subjective self-reports capture myriad markers of sleep disturbance, each offering quantitative and qualitative insights into sleep experiences that may be distal risk factors for GAD symptoms. Actigraphy could capture activity (or movement) counts at rest, sleep, and active wake phases. Wearable actigraphy sensors also measure SOL, TST, sleep efficiency, and wake after sleep onset (WASO), providing consistent observations that usually concur with polysomnography recordings (Smagula et al., 2016; Smith et al., 2018). Subjective self-reports, including sleep diaries, provide essential data on sleep quality appraisals that may or may not correlate highly with actigraphy outcomes (Fabbri et al., 2021; Pierson-Bartel & Ujma, 2024). Both indices are vital in discerning the long-term effects of sleep disturbances, including future GAD symptoms.

Given research, theory, and calls to advance precision psychiatry aims (van Dellen, 2024), the present study used ML to determine objective and subjective sleep disturbance predictors of nine-year GAD symptoms in a community adult sample. Our aims were twofold. First, we expected that the multivariable model, incorporating a high-dimensional set of actigraphy-indexed and self-reported sleep disturbance variables, would predict nine-year GAD symptoms with a meaningful *R*-squared (*R*^*2*^) statistic of at least 10 % (Hypothesis 1; Gao, 2023). Hypothesis 1 is crucial, as an acceptable model fit is a prerequisite for interpreting both linear and nonlinear relationships among predictors and the GAD severity outcome. Second, we hypothesized that actigraphy and self-reported variables capturing various aspects of sleep disturbances would predict higher nine-year GAD severity (Hypothesis 2). Accurately interpreting these relations is essential to theory development. Moreover, our multivariable ML analyses adjusted for relevant clinical and sociodemographic covariates to prevent *p*-hacking and related issues (Fitzpatrick et al., 2024).

## 2. Method

### 2.1. Participants

Community-dwelling adults enrolled in the Midlife Development in the United States (MIDUS) Biomarker project (*N* = 1054) were included in the present secondary data analysis (Ryff et al., 2021, 2019). At Wave 1 (W1; 2004–2006), the mean age of the sample was 55.32 (*SD* = 11.78, range = 34.00–84.00). Regarding gender, whereas 577 (54.7 %) were women, the remaining 477 (45.3 %) were men. Education levels comprised no high school education (51, 4.8 %), completed high school education (238, 22.6 %), some college education (301, 28.6 %), finished college, university, or post-graduate education (214, 20.3 %), and the remaining did not disclose (250, 23.7 %). Racial demographics included White individuals (988, 93.7 %), African Americans, Asian Americans, Native Americans or Pacific Islanders, or other racial identities (66, 6.3 %). The proportions of participants with a GAD diagnosis were 2.09 % (22/1054) and 2.37 % (25/1054) at W1 and W2, respectively.

### 2.2. Eligibility criteria

Inclusion criteria comprised the following: participation in the initial MIDUS project (Brim et al., 2020); self-reported ability to travel for a biomarker data collection protocol with minimal health or logistical risks; voluntary informed consent (Love et al., 2010). Exclusion criteria included medical illnesses that increased travel risks, a recent travel schedule, and shift work patterns. These exclusions maximized the quality of the actigraphy and sleep diaries for community-dwelling adults, ensuring they captured population-level variations in sleep-wake patterns (Aqua et al., 2024; Schreiber & Dautovich, 2019).

### 2.3. Procedures

#### 2.3.1. Overview

All participants completed a biomarker data collection protocol, including a seven-day passive actigraphy wearable assessment at W1 (Love et al., 2010). The W1 assessment also included a survey of subjective sleep disturbances and clinical interviews assessing GAD and related symptoms with the Composite International Diagnostic Interview-Short Form (CIDI-SF; Kessler et al., 1998a; Kessler & Üstün, 2004). The CIDI-SF used herein aligns with the Diagnostic and Statistical Manual of Mental Disorders, Third Edition-Revised (DSM-III-R; Wittchen, 1994). More details are given in the Measures subsection.

#### 2.3.2. Sleep actigraphy protocol

Participants received a sleep actigraphy wearable (Mini Mitter Actiwatch-64 activity monitor) for seven days, which tracked activity movements in active wake, rest, and sleep phases. To standardize the protocol, the actigraphy watches were worn from Tuesday morning at home, following the two-day MIDUS biomarker data collection site visit, until the following Tuesday morning (Kim et al., 2016). Rest and sleep bouts were also captured in the active wake, rest, and sleep phases (Bisson & Lachman, 2023). Participants also completed daily sleep diaries to record the start and end of rest phases (Lee et al., 2025). Daytime naps were not captured by the actigraphy but were recorded in the daily sleep diaries, in which individuals reported the length of each nap (Devine & Wolf, 2016). Comparatively, nighttime patterns were recorded by the actigraphy. Although mood variables were also collected daily (Zainal et al., 2024a), they were not central to the present research aims and thus excluded from the current analyses. These sleep disturbance indices were recorded during the active wake, rest, and sleep phases: activity counts, sleep bouts, sleep efficiency, SOL, TST, WASO, wake bouts, and wake time (counts and percentages; Lee, 2022). Sleep efficiency was computed as the ratio of sleep time in bed, derived from the actigraphy-based sleep epochs. SOL indicated the epoch-based duration between self-reported bedtime and the first sustained sleep period. WASO reflected the total epoch-based awake time between sleep onset and ultimate awakening. TST measured the epoch-based sleep duration from sleep onset to ultimate awakening. The MIDUS project initially referred to TST as “time dozing before rising,” but this label was later changed to TST for greater clarity and consistency with the broader literature.

Additionally, we did not calculate within-person variability indicators (e.g., standard deviation of sleep metrics) for any actigraphy or self-reported sleep indices. The average value of each actigraphy variable was instead computed across the seven-day data collection timeframe. This decision was based on our objective of capturing everyone’s normative sleep-wake and rest-disruption patterns, consistent with our emphasis on trait-level attributes that can predict nine-year GAD severity. Further, adding several variability indices would have expanded the high-dimensional predictor set, potentially exacerbating collinearity and redundancy and compromising model interpretability and predictive accuracy. Therefore, despite their informative value, variability metrics were not included for better alignment with the present hypotheses and the reasons stated here.

Several definitions in the MIDUS project deserve clarification. ‘Activity counts’ is defined as the quantified frequency and intensity of wrist movements in a specific epoch (30 s to 1 min) through a built-in accelerometer (Chung, 2017). Validated algorithms (e.g., Cole-Kripke) identified sleep-wake status were applied to process and record these counts. ‘Sleep bouts’ is operationalized as periods of sleep identified by the algorithms, delimited by wakeful episodes (Devine & Wolf, 2016). A sleep bout is thus referred to as a continuous, uninterrupted period where the individual is estimated to be sleeping, and the counts of such bouts inform about sleep fragmentation and continuity. This method aligns with previous MIDUS applications that processed actigraphy data to yield summary metrics based on activity counts, validated algorithms, and standardized scoring procedures (Lee, 2022; Lee et al., 2025).

### 2.4. Measures

#### W1 and W2 GAD symptoms.

Participants stated if they experienced the following symptoms in the past 12 months linked to worrying about half the days or most days based on the CIDI-SF interview (Kessler et al., 1998a, 1998b): (i) restlessness; (ii) on edge, keyed up, or nervous; (iii) irritability; (iv) concentration issues; (v) memory problems; (vi) low energy; (vii) easily fatigued; (viii) muscle aches or tension. Each item’s score ranged from 1 (*never*) to 4 (*most days*). A dimensional GAD severity score was computed through the summation of all item scores. Two sleep items (trouble falling asleep and staying asleep) were removed to prevent tautological analyses, and sensitivity analyses were conducted while prioritizing the analysis that excluded them. Past research showed that this continuous CIDI-SF GAD scale had good internal consistency (Cronbach’s α =.98 at both W1 and W2 herein), excellent convergent validity, and strong discriminant validity (Ng et al., 2024).

Additionally, the CIDI-SF provided a categorical measure of GAD based on the DSM-III-R criteria. Individuals who expressed worrying excessively almost every day in the past 12 months while experiencing three or more of the above-stated symptoms were diagnosed with GAD. The categorical measure has been shown to have good sensitivity to change and construct validity (Zainal & Newman, 2019).

#### W1 Panic disorder (PD) symptoms.

Given their comorbidity with GAD symptoms (Herr et al., 2014), PD symptoms were included as covariates. Participants similarly responded to the CIDI-SF interview (Kessler et al., 1998a, 1998b) by endorsing if they experienced any of these symptoms linked to cued or non-cued panic attacks or spells in the past 12 months: (i) heart palpitations; (ii) chest or stomach discomfort, pain, or tightness; (iii) sweating; (iv) shaking or trembling; (v) chills or hot flashes; and (vi) sense of unreality with surroundings. Each item was rated as 0 (*absent*) or 1 (*present*). Previous studies suggested that this continuous CIDI-SF PD scale had good internal consistency (α = .86 herein) and strong construct validity (Zainal & Newman, 2022a, 2022b).

#### W1 Major depressive disorder (MDD) symptoms.

As the co-occurrence between MDD and GAD has been well established (Zhou et al., 2017), MDD symptoms were also adjusted for as confounders. Participants responded to the CIDI-SF interview (Kessler et al., 1998a, 1998b) stating whether these MDD symptoms associated with depressed mood or loss of pleasure for at least two weeks were present in the past 12 months: (i) loss of interest; (ii) easily fatigued or atypically low energy; (iii) appetite gain or loss; (iv) trouble falling or staying asleep; (v) concentration issues; (vi) feelings of worthlessness; and (vii) thoughts about death. Each item was recorded as 0 (*absent*) or 1 (*present*). Prior work implied that this continuous CIDI-SF MDD scale showed high internal consistency (α = .85 herein) and good construct validity (Sarkar et al., 2024; Zainal et al., 2024a).

#### W1 Alcohol use disorder (AUD) and substance use disorder (SUD) symptoms.

Prior research has shown that AUD (Ivan et al., 2014) and SUD (Soraya et al., 2022) symptoms could be comorbid with GAD symptoms. AUD symptoms were measured with the Alcohol Screening Test (Selzer, 1971). Participants responded if they had such alcohol use issues in the past 12 months: (i) emotional or psychological disturbances; (ii) strong cravings, desires, or urges that were hard to resist; (iii) at least a month or more of using alcohol or recovering from its impacts; (iv) higher tolerance by using more to attain similar impacts; and (v) increased risks of injury. Each item was coded as 0 (*absent*) or 1 (*present*). Previous studies have implied that the AUD symptom scale has good internal consistency (α = .70 herein) and strong construct validity (Win et al., 2021; Zainal et al., 2024a).

In addition, SUD symptoms were measured on a seven-item scale developed by the MIDUS researchers (Turiano et al., 2012). Participants stated if they experienced these symptoms linked to substance use (e.g., stimulants, prescription painkillers, nerve pills, marijuana, inhalants, hallucinogens) in the past 12 months: (i) increased risks of injury; (ii) heightened consumption than intended; (iii) emotional or psychological disturbances; (iv) excessive time with substance use; (v) higher tolerance such that more substance use is needed to experience similar effects; (vi) consuming larger amounts; and (vii) academic or work impairments. This SUD scale has shown excellent internal consistency (α = .81 herein) and good construct validity (Turiano et al., 2012; Zainal et al., 2024a).

#### W1 Pittsburgh Sleep Quality Index (PSQI; Buysse et al., 1989).

The PSQI measured seven domains of self-reported sleep disturbances in the past month: (i) daytime dysfunction; (ii) habitual sleep inefficiency; (iii) sleep disturbances; (iv) sleep duration; (v) SOL; (vi) sleep medication consumption; and (vii) subjective poor sleep quality. The PSQI scores have shown acceptable internal consistency (α = .70 herein) and good construct validity with other insomnia scales (Carpenter & Andrykowski, 1998). Details on PSQI cut-points and the proportion of participants who met each cut-point are in the online supplemental materials (OSM).

#### W1 Pharmacological and psychological treatment.

Pharmacological treatment was defined as self-reported medication use counts in the past 30 days for any of the following reasons: anxiety/depression, arthritis, birth control, cholesterol, diabetes, headaches, heart condition, hormone therapy, lungs, pain, or ulcer (Ryff et al., 2021). Psychological treatment was measured with a dimensional scale that counted the number of mental health professionals (e.g., counselors, psychiatrists, psychologists) participants visited in the past 12 months. Medical treatment utilization was operationalized as the self-reported counts of visits participants had with medical professionals for check-ups, routine appointments, or urgent care in the past 12 months.

### 2.5. Data analyses

All analyses were conducted using the *R* software coding environment (version 4.4.2) for multivariable ML analyses (R Core Team, 2025). Across multiple indicators, the associations between actigraphy-indexed sleep markers (e.g., total activity counts, sleep efficiency, WASO) and PSQI self-reported dimensions (e.g., daytime dysfunction, sleep disturbances) were small overall. Table S1 in the OSM presents the descriptive statistics of all sleep disturbance variables of interest, as well as the clinical and demographic covariates included in the predictor set, which comprised 44 variables. Table S2 presents the correlation matrix of associations between actigraphy and self-report sleep markers. Most correlation coefficients fell below *r* = .20. These small associations highlight that actigraphy and self-report assess distinct perspectives on sleep, reflecting objective rest-activity patterns rather than subjective appraisals of sleep quality. Therefore, testing these markers separately is critical to distinguish their distinct contributions in predicting nine-year GAD severity.

Feature engineering steps were carried out independently in the training and testing folds before the multivariable ML prediction analyses to avoid data leakage (James et al., 2013). Normalization was conducted on continuous variables by standardizing values to a mean of 0 and a standard deviation (*SD*) of 1. One-hot encoding was applied to nominal variables. Missing data in the predictor set of the training folds were handled using random forest (RF) imputation, which was superior to multiple imputation by accounting for any existing interactions and nonlinearities (Shah et al., 2014), as implemented in the *missRanger* package (Mayer, 2024).

To evaluate the multivariable ML predictive models (Hypothesis 1), we conducted a nested cross-validation (NCV) approach (Lewis et al., 2024). This method enables the optimization of model parameters and the attainment of unbiased predictive performance estimates. Essentially, the NCV framework comprises inner-fold loops for model selection, training, and tuning, as well as outer-fold loops to assess the extent to which the tuned model generalizes to unseen data. This method used a random forest (RF) algorithm, a commonly used ensemble approach that aggregates predictions from multiple decision trees to improve precision and robustness.

Our RF algorithm within the NCV framework was conducted using the *caret* (Kuhn, 2008) and *ranger* (Wright & Ziegler, 2017) packages. An important step in optimizing an ML model is hyperparameter tuning, typically done with the *expand.grid* function for tree-based ML algorithms. We systematically varied the tuning grid by altering these three parameters: (i) *splitrule* (the procedures for making choices to split decision tree nodes); (ii) *mtry* (the random decision of the number of predictors to retain at each split); and (iii) *min.node.size* (the minimum observations or sample size in a terminal node). These parameters influence how the model strikes a balance between bias and variance (Breiman, 2001). Based on recommendations, we aggregated the estimated prediction scores across numerous decision trees to yield a final prediction score (Namamula & Chaytor, 2022).

To provide context for the RF performance, six other multivariable ML algorithms were tested (Pargent et al., 2023). These algorithms included linear models: least absolute shrinkage and selection operator (LASSO), ridge regression, and elastic net regression. The algorithms also included two other tree-based models (decision trees and gradient boosting machine [GBM]) and the support vector machine (SVM) that could capture both linear and nonlinear associations. More details about the tested ML models are expanded in the OSM.

For each tested ML algorithm, both the inner-loop folds (model selection, training, and hyperparameter tuning) and the outer-loop folds (model testing) were set to 5. To assess the accuracy and stability of predictive performance estimates, we carried out 1000 bootstrap resampling iterations (Kosko et al., 2024). We calculated the 95 % confidence intervals (CIs) for three conventional regression performance indices, which are grounded in best practices: (i) root mean square error (RMSE); (ii) mean absolute error; (iii) *R*^2^ metric. Model calibration plots were also constructed to test the degree to which predicted scores corresponded with observed scores (Huang et al., 2020).

To improve the interpretability of predictor-outcome associations (Hypothesis 2), we implemented two interpretable ML (also called explainable artificial intelligence [XAI]) methods: partial dependence plots (PDPs) and Shapley additive explanations (SHAP; Molnar, 2022). PDPs compute the mean change in the predicted outcome value as a single predictor variable changes while keeping the remaining variables constant (Kyriazos & Poga, 2024). SHAP values, derived from game-theoretic principles, quantify the contribution of each predictor variable to each prediction at the participant level (Lundberg & Lee, 2017).

Although PDPs offer the intuitive appeal of average predictor effects, SHAP builds on its logic by estimating predictor importance rankings, interactions, and nonlinear associations. In particular, we utilized SHAP bee swarm plots, which illustrate the sign and magnitude of each predictor’s contribution across individuals, while highlighting the most impactful predictors (Kovač et al., 2024; Lundberg et al., 2020). Collectively, PDPs and SHAP offer insights into complex ML algorithms, such as RF, enabling the transformation of “black box” ML models into intuitive instruments.

## 3. Results

### 3.1. Main findings (Hypothesis 1)

#### Hypothesis 1.

assessed how the multivariable ML models could constructively forecast W2 GAD severity. Table 1 presents the performance metrics of the predictive model for all algorithms. The RF algorithm yielded the best performance across all performance metrics, indicating that it successfully captured the complex associations in the W1 data. Note that whereas RMSE and MAE measure the average prediction errors (lower is better), *R*^*2*^ represents the percentage of variance accounted for (higher is better). The RF model yielded the lowest RMSE (0.050, 95 % CI [0.047–0.053]) and MAE (0.038, 95 % CI [0.036–0.040]), indicating stronger predictive accuracy than the other algorithms. In addition, it achieved the largest *R*^*2*^ value (69.7 % [67.3 %–71.9 %]), indicating that it accounted for more than half of the variance in W2 GAD severity.

Calibration plots (Figure S1 in the OSM) were also examined to assess the degree to which predicted values corresponded with actual scores. Calibration is defined as the extent to which the model’s forecasted scores match real-world outcome values. Error-based indices for the calibration analyses were consistently close to 0 (MAE: 0.079; RMSE: 0.060; *R*^*2*^ metric: 25.6 %), indicating good calibration. Together, these results offer support for Hypothesis 1.

### 3.2. Predictor-outcome relations (Hypothesis 2)

#### Hypothesis 2.

**Fig. 2.**
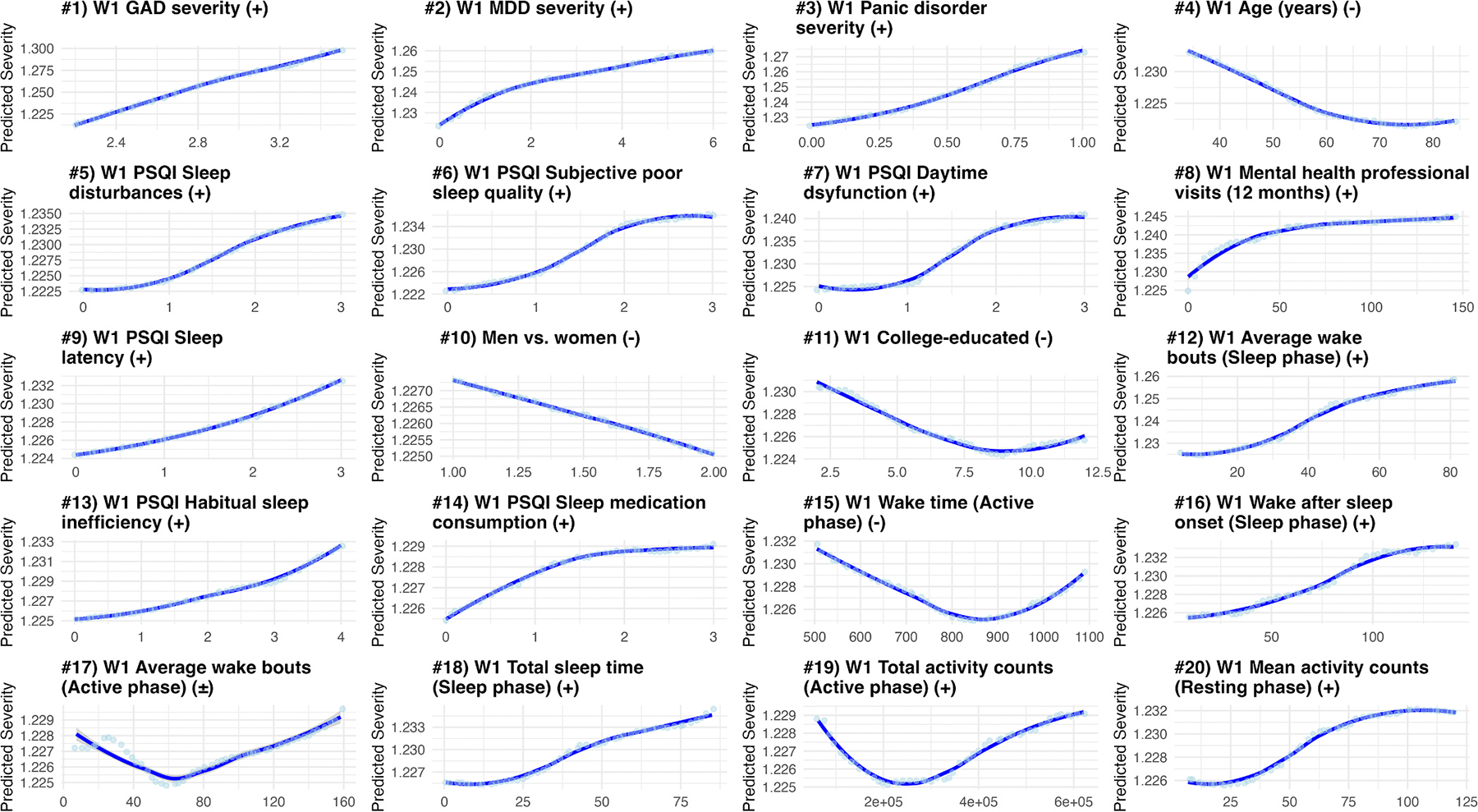
Partial dependence plots of W1 sleep disturbance variables predicting W2 GAD severity. *Note.* W1, wave 1 (2004–2006); W2, wave 2 (2013–2014); GAD, generalized anxiety disorder; MDD, major depressive disorder; PSQI, Pittsburgh Sleep Quality Index.

**Fig. 3.**
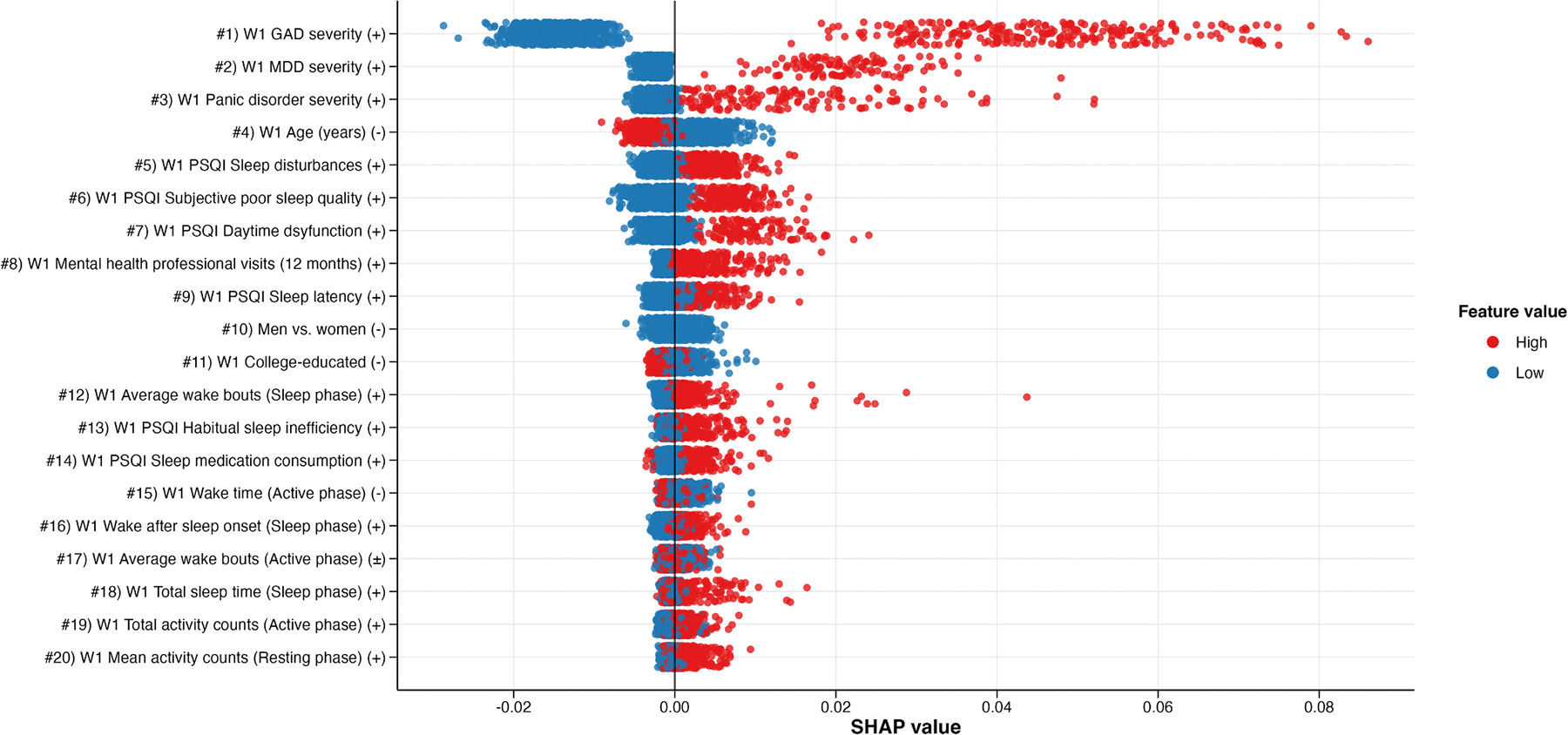
SHAP bee swarm plot of W1 sleep disturbance variables predicting W2 GAD severity. *Note.* SHAP, Shapley additive explanations; W1, wave 1 (2004–2006); W2, wave 2 (2013–2014); GAD, generalized anxiety disorder; MDD, major depressive disorder; PSQI, Pittsburgh Sleep Quality Index. Each data point represents participant-level data. Red data points denoted positive predictor-outcome relations, whereas blue data points denoted negative relations, aggregated into a density plot for each predictor to depict global and local participant-level conditional predictor effects on the outcome.

examined the pattern of associations between W1 variables and W2 GAD severity. To enhance interpretability, we employ two visualization approaches typical in XAI: PDPs (Fig. 2) and SHAP bee swarm plots (Fig. 3). PDPs display the mean association between a predictor variable and the outcome. Comparatively, SHAP plots offer a ranked order of variable importance that captures both linear and nonlinear patterns as well as higher-order interactions.

#### 3.2.1. Main patterns

Self-reported sleep disturbances ranked highly in predicting W2 GAD severity (# indicated relative importance): more sleep disturbances (#4), subjective poor sleep quality (#5), longer sleep latency (#6), daytime dysfunction (#7), greater habitual sleep inefficiency (#10), and sleep medication consumption (#18). Two actigraphy-indexed rest phase variables ranked highly in predicting W2 GAD severity: higher maximum (#12) and total (#13) activity counts. Only one sleep phase variable–longer TST (#14)–and one active phase variable–higher average sleep bouts (#20)–predicted higher W2 GAD severity.

#### 3.2.2. Sensitivity analyses

These patterns remained after adjusting for these covariates: GAD severity (#1), panic disorder severity (#2), MDD severity (#3), age (#8), mental health professional visits (#9), gender (#16), past 30-day medication counts (#17), and past 12-month medical professional visits (#17). Sensitivity analyses aligned with the initial findings when sleep was added to the GAD symptom severity measures (Table S3; Figures S2–S4). Collectively, these outcomes supported Hypothesis 2, affirming that both actigraphy-based and self-reported sleep disturbances were essential predictors of W2 GAD severity.

## 4. Discussion

The present study used recommended ML methods to identify the objective and subjective sleep disturbance predictors of nine-year GAD symptoms. In support of Hypothesis 1, our multivariable predictive ML model showed strong predictive performance, as indicated by the *R*^*2*^ value, with narrow 95 % CIs (69.7 % [67.3 %–71.9 %]), offering confidence in the estimates (Gao, 2023). Findings also aligned with Hypothesis 2, revealing that various indices of objective actigraphy and subjective self-reported sleep disturbance variables consistently predicted higher nine-year GAD severity. The relative importance of subjective over objective sleep disturbance predictors of nine-year GAD symptoms extended data that subjective indices had precedence in mediating the relation between pre-sleep worrying and later emotional well-being (Werner et al., 2025).

Our findings of W1 sleep disturbances preceding and predicting higher W2 GAD severity concur with cognitive-behavioral and neurobiological frameworks that posit sleep to both trigger and maintain anxiety disorders. Cognitive theories suggest that daytime dysfunction, poorer subjective sleep quality, and prolonged sleep latency can exacerbate attentional biases toward threats, increase worry, and reinforce safety behaviors (e.g., lengthening the time spent in bed; Akram et al., 2023; Harvey, 2008; Harvey & Greenall, 2003). These factors inevitably perpetuate cyclical experiences of arousal and sleep fragmentation. The transdiagnostic model expands on cognitive theories by contextualizing sleep disturbances as a common risk mechanism across unique mental disorders (Harvey et al., 2011). Increased daytime and nighttime arousal vis-à-vis issues with the modulation of cognitive-emotional load would ` aggravate and prolong GAD symptoms. Neurobiologically, the hyperarousal framework suggests that discontinuities in rest or sleep phases, as well as hypersomnia (characterized by longer TST), indicate poor regulation of sleep-wake modulation systems. Such dysregulations typically manifest as persistent activation of the autonomic, cortical, and hypothalamic-pituitary-adrenal (HPA) axis (Riemann et al., 2010). Moreover, somatic dysregulation both fragments sleep and interacts reciprocally with worry-related threat hypervigilance, consistent with data showing that impaired architectures in rapid eye movement (REM) and non-REM (NREM) stages can worsen such psychopathological features (Papadimitriou & Linkowski, 2005; Uhde et al., 2009). Collectively, these models might account for the observed pattern in which self-reported and actigraphy-based indices of sleep disturbances serve as long-term risk factors for higher GAD severity, above and beyond clinical and demographic covariates.

The observed outcomes, both actigraphy and self-reported markers of sleep disturbances, predicted nine-year GAD severity, aligning with and building on previous research across clinical and research samples. Aligned with Tsypes et al. (2013), self-reported indices (e.g., daytime dysfunction, prolonged sleep latency) meaningfully predicted future GAD severity, highlighting that sleep fragmentation markers are not solely a correlate but may function as key risk factors. Further, our results concurred with actigraphy research showing that altered sleep continuity or timing, especially longer TST, and higher activity during rest phases, are associated with elevated worry severity (Carbone et al., 2023; Peng et al., 2024b). Crucially, by combining actigraphy and self-report data, our findings buttressed the assertions of Ivan et al. (2014) and Nguyen et al. (2022) that objective and subjective assessments provide unique yet complementary perspectives on the sleep-worry connection. The observed outcomes reinforce prospective interpretations from adolescents and adults, demonstrating that baseline sleep disturbances forecast future anxiety above and beyond depressive symptoms (Peng et al., 2024a; Tsypes et al., 2013). As in prior research, our study precludes strong causal inferences (Blackwell & Glynn, 2018), and residual confounding (e.g., emotion dysregulation, recent stressors) might partly explain our findings. However, longitudinal sleep-GAD severity effects remained after controlling for various covariates. This outcome may indicate that the identified sleep disturbance indices are reliable risk factors for long-term GAD severity.

Furthermore, it is plausible that distinct sleep components interact to influence pathways associated with GAD risk. For example, longer TST coupled with reduced sleep efficiency or increased physical activity during rest phases may worsen hyperarousal experiences and excessive worry. Simultaneously, deviations in both active and rest-phase activity counts might worsen circadian misalignment. Future prospective studies should assess such interaction effects to determine how actigraphy-indexed sleep disturbances confer additive or multiplicative risk for nine-year GAD symptom severity.

In the GAD-related literature, both actigraphy-indexed and self-reported sleep-wake indices provide unique and complementary perspectives on the conceptualization of circadian and sleep disturbances. Actigraphy provides objective, naturalistic indicators of circadian rhythms, physical activity, sleep duration, and efficiency that can identify discontinuities in sleep or rest phases, which may be missed by self-report. Examples include how deficits in gross motor activity and vigorous physical activity are more pronounced in individuals with comorbid anxiety and depression (Difrancesco et al., 2019). In community adults, actigraphy-indexed data further signal the importance of sleep fragmentation as a strong marker of anxiety disorders independent of other circadian variables, emphasizing how dysregulated physical activity may partly explain worry-sleep relations (Luik et al., 2015). Experimental data in GAD samples also showed that exercise programs, especially resistance training, enhance actigraphy-based and self-reported sleep outcomes (e.g., decreased SOL, improved sleep efficiency), thereby improving clinical outcomes (Herring et al., 2015). Self-reports, on the other hand, may simultaneously capture subjective stress severity and fluctuations in subjective insomnia symptoms, which can be misaligned with actigraphy data (Difrancesco et al., 2019). Nonetheless, despite promising findings from prior reports and the current study, reviews indicate that actigraphy remains underutilized in the anxiety literature (Pastre & Lopez-Castroman, 2022). The dearth of prospective studies and methodological variability compounds this issue, limiting its present utility in measurement-based care and routine outcome monitoring. Collectively, our findings and prior research emphasize the importance of combining subjective self-reports with objective actigraphy to holistically study the role of sleep disturbances as both distal risk factors and targets for assessment and treatment.

## 5. Limitations and strengths

The present study outcomes should be interpreted with consideration of several limitations. First, future studies should include unmeasured potential confounds, including factors related to circadian clock genes (Bauducco et al., 2020). Second, and related, subsequent research should add polysomnography indices (Galbiati et al., 2018) into the multivariable predictor set to enrich the detection of the relative importance of multimodal sleep disturbance variables. Third, although the DSM-III-R and DSM-5 definitions of GAD symptoms were similar to a large degree (Abel & Borkovec, 1995; Brown et al., 1995; Starcevic & Portman, 2013), replication efforts should use the latest psychometrically validated assessments. Fourth, replication attempts should recruit more diverse and representative samples to improve the generalizability of findings, given that 93.7 % of the sample identified as White. Fifth, external validation using an independent community-adult sample would increase confidence in the inferences (Cabitza et al., 2021). Sixth, although the rest-activity disruption model of worry posits that the relationship between sleep disturbances and GAD symptoms is complex and bidirectional (Nguyen et al., 2022), the present study focused on the prognostication of long-term GAD symptoms. Seventh, our observations apply to the between-person level and may thus not extend to the within-person domain in both strength and sign, which is critical to remember to prevent the ecological fallacy (Curran & Bauer, 2011). Intensive longitudinal design work is required to assess the degree to which the findings generalize to within-person processes (Molenaar, 2004). Finally, although the ML models detected patterns between W1 variables and W2 GAD severity, strong causal inferences cannot be made without experimental studies or more thorough causal inference analyses. W1 variables identified as distal risk factors for higher W2 GAD severity may still capture correlations from common correlates or unmeasured confounders, warranting caution in interpreting these findings for prevention and treatment purposes (Brand et al., 2023; Leist et al., 2022). However, the study’s strengths included a well-powered adult community sample, a robust nested cross-validation ML approach, and the use of a large set of sleep disturbance predictors, including objective and subjective measures.

## 6. Clinical implications

When extrapolating clinical implications, it is essential to consider that our longitudinal study employed observational methods, which preclude strict causal inference and may lead to residual confounding, despite extensive covariate adjustment (Hernán & Robins, 2023). To assess the potential implications of sleep disturbances for the prevention and treatment of long-term GAD symptoms, future studies should employ designs that assess bidirectionality, mediation, and portable patterns. First, intensive longitudinal designs, possibly through ecological momentary assessments (EMA), are required to clarify within-person trajectories. Multimodal measures that combine actigraphy, daily diary, and EMAs should capture arousal, worry, and related GAD constructs. Such EMA data could then be analyzed using within-person methods, such as dynamic structural equation modeling (Rodebaugh et al., 2022) and network-based multilevel vector autoregressions (Haslbeck et al., 2021). Second, more experiments and efficacy trials of scalable cognitive-behavioral therapy for insomnia (Zainal et al., 2024b), structured physical exercise (Xie et al., 2021), and mindfulness training (Kennett et al., 2021) should be conducted to facilitate causal inferences. Follow-up mediational analyses are key for uncovering plausible treatment mechanisms, such as executive functioning and hyperarousal. Moderators, such as age and sex, should be explored to advance precision mental health. Third, external validation (Collins et al., 2024) is essential for assessing the generalizability and transportability of our multivariable models, a step toward creating actionable prognostic tools. Last, implementation science work is required to evaluate cost-effectiveness and equity in various clinic, community, and corporate settings (Zainal et al., 2025). If these studies provide converging evidence that within-person reductions in sleep disturbances precede and predict reductions in worry, routine monitoring of both objective and subjective sleep disturbances within a stepped-care framework could be recommended. Until then, any plausible clinical implication should be interpreted as hypothesis-generating instead of a recommendation.

## 7. Conclusion

Among a large community-adult sample, a nested cross-validation multivariable model integrating actigraphy and self-report markers of sleep disturbances accurately predicted nine-year GAD symptoms. Interpretable ML methods identified key markers of sleep disturbances. These outcomes underscore the predictive value of both subjective and objective sleep disturbance indices. However, these patterns yielded inferences at the between-person rather than within-person level, hindering strong causal inferences and potentially concealing essential within-person processes. Future work building on these findings should leverage intensive longitudinal designs and suitable within-person methods, with an analytic pipeline that includes experimental methods and external validation procedures. If similar patterns are replicated within persons and across diverse settings, the development of actionable prognostic calculators implemented within stratified care contexts would be justified.

## Supplementary Material

1

## Figures and Tables

**Fig. 1.**
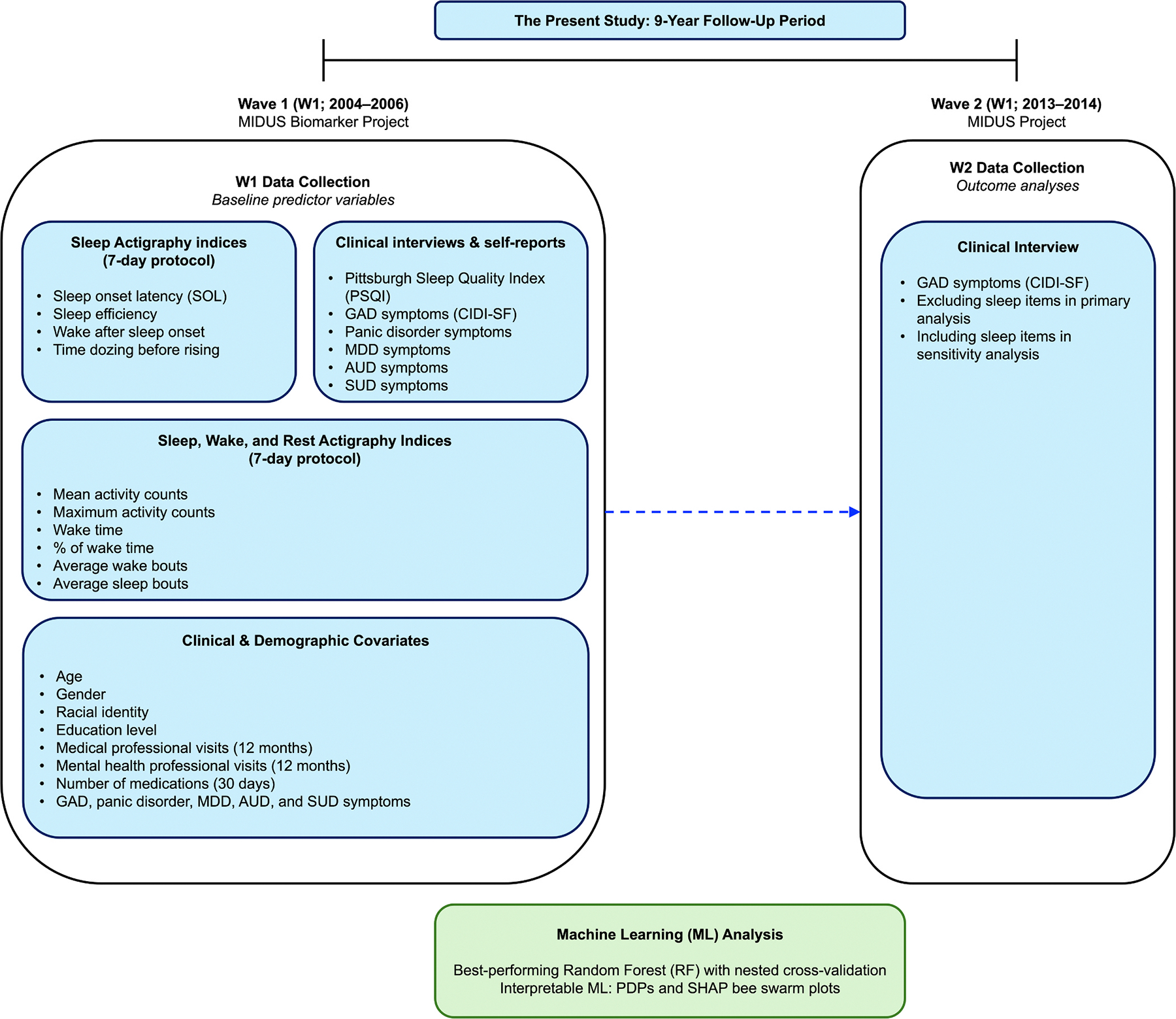
Summary of the overall MIDUS project with specific variables collected at each assessment wave *Note.* MIDUS, Midlife Development in the United States; CIDI-SF, Composite International Diagnostic Interview-Short Form; GAD, generalized anxiety disorder; MDD, major depressive disorder; AUD, alcohol use disorder; SUD, substance use disorder; PDPs, partial dependence plots; SHAP, Shapley additive explanations.

**Table 1:** Multivariate ML model performance metrics of W1 variables predicting W2 GAD severity.

Metric	Estimate	LCI	UCI

Least absolute shrinkage and selection operator (LASSO)			
RMSE	0.078	0.074	0.082
MAE	0.055	0.052	0.059
*R* ^2^	0.277	0.215	0.341
Ridge regression			
RMSE	0.078	0.074	0.082
MAE	0.056	0.053	0.060
*R* ^2^	0.271	0.206	0.338
Elastic net regression (ENR)			
RMSE	0.078	0.074	0.082
MAE	0.055	0.052	0.059
*R* ^2^	0.274	0.211	0.342
Decision trees (DCT)			
RMSE	0.076	0.072	0.081
MAE	0.054	0.050	0.057
*R* ^2^	0.304	0.238	0.369
Random forest (RF)			
RMSE	0.050	0.047	0.053
MAE	0.038	0.036	0.040
*R* ^2^	0.697	0.673	0.719
Gradient boosting machine (GBM)			
RMSE	0.068	0.064	0.072
MAE	0.049	0.046	0.052
*R* ^2^	0.440	0.384	0.499
Support vector machine (SVM)			
RMSE	0.082	0.077	0.088
MAE	0.051	0.047	0.055
*R* ^2^	0.222	0.156	0.293

*Note.* ML, machine learning; W1, wave 1 (2004–2006); W2, wave 2 (2013–2014); GAD, generalized anxiety disorder; LCI, lower bound of the 95 % confidence intervals (CIs) of the bootstrapped model performance metric; UCI, upper bound of the 95 % CIs of the bootstrapped model performance metric; RMSE, root mean squared error; MAE, mean absolute error; *R*^2^, *R*-squared.

## Data Availability

The authors do not have permission to share data.

## Appendix A. Supporting information

Supplementary data associated with this article can be found in the online version at doi:10.1016/j.janxdis.2025.103095.
