# Supplementary material for: Actigraphy and subjective sleep predictors of nine-year generalized anxiety disorder
Source: J Anxiety Disord. Author manuscript; Available in PMC 2026 Jul 25. (PMC13401219; doi:10.1016/j.janxdis.2025.103095)
Supplement: 1 [file NIHMS2186733-supplement-1.docx]

# Online Supplemental Materials (OSM)

## PSQI Cut-Offs and Proportion of ‘Poor’ and ‘Good’ Sleepers

The Pittsburgh Sleep Quality Index (PSQI) self-report assessment has been investigated for its psychometric validity in measuring sleep quality, where a cut-point of PSQI total score > 5 has been regarded as the boundary to differentiate ‘poor’ from ‘good’ sleepers in diverse populations (Buysse et al., 1989; Shahid et al., 2011). The original PSQI study observed good sensitivity (89.6%) and specificity (86.5%) with a cut-off of > 5, which continues to be applied in both the general population and psychiatric samples (Carpi, 2025). Nonetheless, recent studies highlighted the relevance of population-based cut-points. For example, in patients with ischemic stroke, a PSQI ≥ 9 cut-point yielded excellent psychometric performance (area under the receiver operating characteristic curve [AUC] = .960, sensitivity = 100%, specificity = 85.2%). Likewise, among college students and other target samples, alternative cut-points, such as 6 or 7, have been recommended to optimize the diagnostic precision based on the assessment aims (Dietch et al., 2016; Donahue et al., 2025). Therefore, although the PSQI > 5 cut-point stays as the standard reference, sensitivity analyses with higher and lower scores are recommended.

Given this rationale, we examined the proportion of ‘poor’ and ‘good’ sleepers using various cut-offs. For the standard PSQI ≥ 6 cut-point, the distribution of ‘poor’ and ‘good’ sleepers was 46.6% and 53.4%, respectively. For the PSQI ≥ 7, ≥ 8, and ≥ 9 cut-points, the proportions of poor sleepers were 38.3%, 29.7%, and 20.6%, respectively.

## Machine Learning (ML) Models in the Present Study

**Least absolute shrinkage and selection operator (LASSO):** The LASSO is an ML approach that assists with the prediction of outcomes, including generalized anxiety disorder (GAD) severity, by automatically identifying solely the top essential data from a high-dimensional set of predictors (Brand et al., 2023). These processes are conducted by imposing a penalty on complex multivariable models, making all variables shrink toward 0, especially variables of 0 or weaker predictive importance, thereby simplifying models to enhance interpretability. By honing in on the top-predictor variables, LASSO decreases noise and improves signal, thus aiding clinicians and researchers in constructing enhanced, reliable tools to comprehend and predict mental health outcomes.

**Ridge regression:** Ridge regression is an ML approach that enhances prediction precision when managing multicollinear predictor variables, such as several symptom scores or risk markers in clinical psychological research (Lavelle-Hill et al., 2025). By imposing minimal penalty on highly complex multivariable models, it retains the effect of each predictor in a more balanced way and safeguards against the model concentrating excessively on small details or noise in the observations. To this end, ridge regression generates predictors in more consistent ways by identifying the complex patterns frequently observed in mental health outcomes.

**Elastic net regression (ENR):** ENR is an ML approach that integrates the advantages of both LASSO and ridge regression to enhance predictions, which makes it particularly helpful when dealing with collinear baseline predictors (Li et al., 2021). Drawing a balance between these two penalty types, it chooses the most essential predictors while simultaneously managing multicollinearity, providing consistency and flexibility for complex observations. These benefits render ENR advantageous in predicting mental health outcomes, given how it balances optimally between incorporating adequate essential information and preventing the model from including excessive or irrelevant information.

**Decision trees (DCT):** DCT is a specific ML approach that predicts outcomes by breaking down complex choices into a sequence of “yes or no” decision rules that resemble a schematic flowchart (Kovač et al., in press). During each step of the sequence, the algorithm decides the most critical factor to inquire about (e.g., symptom), and it continues to split the data into smaller sets repeatedly until it arrives at an ultimate prediction (e.g., probability of a mental health outcome). As the schematic decision flowchart is intuitive, DCTs are often employed in psychopathology studies to visualize how unique information sources arrive at distinct predictions explicitly.

**Random forest (RF):** RF is an ML approach that enhances prediction accuracy by integrating the outcomes of unique decision trees with stopping rules, each constructed with random subsets of predictors and observations (Lavelle-Hill et al., in press). This “forest” of DCTs takes an aggregate mean of all predictions (called “votes” in the broader ML literature), which minimizes errors and maximizes model reliability and stability, particularly when managing complex patterns observed in psychopathology data sets. As RF harnesses various questions for each DCT, the odds of it being deceived by atypical data points are lower, and it thus provides more reliable predictions for mental health outcomes.

**Gradient boosting machine (GBM):** GBM is a potent ML algorithm that is created through an iterative sequence of DCTs, wherein each new RCT prioritizes rectifying the errors of the prior RCTs, logically enhancing the global prediction (Kyriazos & Poga, 2024). By “boosting” previous weak DCT learners, GBMs may optimally model complex patterns observed in the data set, rendering them well-suited to comprehend the subtle yet impactful predictors of mental health outcomes. This sequential step-by-step method may improve accuracy, even in the face of highly complex data, as is frequently observed in mental health research.

**Support vector machine (SVM):** SVM is a distinct ML algorithm that divides data, such as symptom scores, into classes by figuring out the optimal “boundary line” that maximizes the length between different classes (David et al., 2025). SVM attends to the hardest-to-categorize cases, called “support vectors,” thereby enhancing prediction accuracy even with complex observations or hard-to-categorize cases. This mathematical method is particularly useful in forecasting mental health outcomes as it manages both simple and complex patterns, facilitating the identification of disordered vs. non-disordered cases.

Table S1

*Descriptive statistics of variables in the W1 variables to predict W2 GAD severity*

|  | *M/n* | (*SD*)/(%) | Minimum | Maximum | Skewness | Kurtosis |
| --- | --- | --- | --- | --- | --- | --- |
| W1 Age (years) | 55.32 | (11.78) | 34.00 | 84.00 | 0.30 | -0.67 |
| Men vs. women | 477 | (45.26) | – | – | – | – |
| W1 College-educated | 214 | (20.31) | – | – | – | – |
| White | 988 | (93.73) | – | – | – | – |
| W1 MDD severity | 0.85 | (2.34) | 0.00 | 14.00 | 2.82 | 7.36 |
| W1 GAD severity | 11.69 | (6.85) | 8.00 | 32.00 | 1.48 | 0.53 |
| W1 Panic disorder severity | 0.43 | (1.18) | 0.00 | 6.00 | 2.90 | 7.84 |
| W1 AUD severity | 0.07 | (0.37) | 0.00 | 4.00 | 7.03 | 56.43 |
| W1 SUD severity | 0.61 | (1.89) | 0.00 | 18.00 | 4.11 | 21.71 |
| W1 PSQI Daytime dysfunction | 0.81 | (0.67) | 0.00 | 3.00 | 0.49 | 0.21 |
| W1 PSQI Habitual sleep inefficiency | 0.72 | (1.16) | 0.00 | 4.00 | 1.47 | 0.90 |
| W1 PSQI Sleep disturbances | 1.28 | (0.56) | 0.00 | 3.00 | 0.61 | 0.47 |
| W1 PSQI Sleep duration | 0.78 | (0.75) | 0.00 | 3.00 | 0.82 | 0.51 |
| W1 PSQI Sleep latency | 0.88 | (0.92) | 0.00 | 3.00 | 0.86 | -0.12 |
| W1 PSQI Sleep medication consumption | 0.57 | (1.07) | 0.00 | 3.00 | 1.57 | 0.79 |
| W1 PSQI Subjective poor sleep quality | 0.97 | (0.68) | 0.00 | 3.00 | 0.46 | 0.52 |
| W1 Mean activity counts (Resting phase) | 31.44 | (18.88) | 6.90 | 120.42 | 1.82 | 4.02 |
| W1 Maximum activity counts (Resting phase) | 683.04 | (207.62) | 201.43 | 1420.33 | 0.83 | 0.82 |
| W1 Wake time (Resting phase) | 69.01 | (34.19) | 22.33 | 234.07 | 1.38 | 2.38 |
| W1 % of wake time (Resting phase) | 14.89 | (7.38) | 5.50 | 41.96 | 1.51 | 2.22 |
| W1 Average wake bouts (Resting phase) | 38.15 | (13.52) | 10.71 | 95.71 | 1.13 | 2.59 |
| W1 Average sleep bouts (Resting phase) | 12.50 | (6.06) | 3.29 | 81.16 | 4.32 | 35.83 |
| W1 Total activity counts (Sleep phase) | 7713.94 | (4604.40) | 1002.33 | 28696.57 | 1.76 | 4.20 |
| W1 Mean activity counts (Sleep phase) | 18.66 | (12.03) | 2.59 | 74.10 | 1.92 | 4.39 |
| W1 Maximum activity counts (Sleep phase) | 510.46 | (176.68) | 185.43 | 1297.33 | 0.91 | 1.30 |
| W1 Average sleep onset latency (Sleep phase) | 25.77 | (23.08) | 0.21 | 128.57 | 1.87 | 3.58 |
| W1 Total sleep time (Sleep phase) | 13.46 | (14.94) | 0.50 | 84.92 | 2.28 | 5.61 |
| W1 Sleep efficiency (%) (Sleep phase) | 81.61 | (9.57) | 44.27 | 93.61 | -1.52 | 2.50 |
| W1 Wake after sleep onset (Sleep phase) | 45.57 | (21.87) | 8.83 | 139.86 | 1.33 | 2.51 |
| W1 Wake time (Sleep phase) | 45.65 | (22.13) | 8.83 | 139.86 | 1.37 | 2.74 |
| W1 % of wake time (Sleep phase) | 10.79 | (5.69) | 2.28 | 31.08 | 1.49 | 2.06 |
| W1 Average wake bouts (Sleep phase) | 32.06 | (10.59) | 10.14 | 71.33 | 0.59 | 0.13 |
| W1 Average sleep bouts (Sleep phase) | 14.25 | (7.43) | 4.26 | 81.44 | 4.37 | 32.35 |
| W1 Total activity counts (Active phase) | 328289.45 | (105549.46) | 62573.67 | 620944.17 | 0.36 | -0.01 |
| W1 Mean activity counts (Active phase) | 335.93 | (108.24) | 65.49 | 660.60 | 0.42 | 0.29 |
| W1 Maximum activity counts (Active phase) | 1376.26 | (364.51) | 429.83 | 2406.83 | 0.38 | -0.10 |
| W1 Wake time (Active phase) | 818.20 | (105.14) | 504.92 | 1085.75 | -0.49 | 0.07 |
| W1 % of wake time (Active phase) | 83.23 | (9.21) | 48.62 | 97.64 | -1.02 | 0.94 |
| W1 Average wake bouts (Active phase) | 62.58 | (30.14) | 7.33 | 158.17 | 0.58 | -0.14 |
| W1 Average sleep bouts (Active phase) | 2.73 | (1.46) | 1.22 | 19.29 | 7.31 | 77.14 |
| W1 Medical professional visits (12 months) | 3.65 | (4.05) | 0.00 | 57.00 | 4.58 | 39.39 |
| W1 Mental health professional visits (12 months) | 2.17 | (8.87) | 0.00 | 145.00 | 9.20 | 110.54 |
| W1 Medications (30 days) | 1.42 | (1.50) | 0.00 | 9.00 | 1.19 | 1.43 |
| W2 GAD severity | 11.71 | (6.89) | 8.00 | 32.00 | 1.46 | 0.46 |

*Note.* W1, wave 1 (2004–2006); W2, wave 2 (2013–2014); MDD, major depressive disorder; GAD, generalized anxiety disorder; AUD, alcohol use disorder; SUD, substance use disorder; PSQI, Pittsburgh Sleep Quality Index. The descriptive statistics for total sleep time (TST) in the sleep phase do not reflect hours directly. Instead, they represent the accrual of TST expressed in units aligned with epoch units in the 7-day actigraphy protocol. In the Midlife Development in the United States (MIDUS) project, an “epoch” is defined as a 30-second interval used by the Actiwatch. Thus, the values of TST herein reflected the total epochs coded as “sleep” during the entire 7-day actigraphy period.

Table S2

*Correlation matrix of the PSQI self-report and actigraphy measures of sleep at W1*

| Variable | 2 | 3 | 4 | 5 | 6 | 7 | 8 | 9 | 10 | 11 | 12 | 13 | 14 | 15 | 16 | 17 | 18 |
| --- | --- | --- | --- | --- | --- | --- | --- | --- | --- | --- | --- | --- | --- | --- | --- | --- | --- |
| 1. W1 PSQI Sleep latency | 0.025 | .235* | 0.052 | .260* | -.119* | .178* | .152* | .104* | .149* | .148* | .138* | .096* | -0.043 | .434* | .372* | .289* | .246* |
| 2. W1 Average SOL (Sleep phase) | – | .088* | .213* | 0.055 | -.577* | .368* | .437* | .192* | .377* | .388* | .455* | .299* | -.216* | .084* | .083* | .061* | .094* |
| 3. W1 PSQI Sleep duration |  | – | 0.019 | .375* | -.074* | 0.057 | .092* | .069* | 0.007 | 0.002 | 0.058 | -.076* | 0.05 | .414* | .146* | .069* | .265* |
| 4. W1 TST (Sleep phase) |  |  | – | 0.029 | -.489* | .331* | .343* | .190* | .360* | .374* | .427* | .330* | -.184* | .066* | 0.055 | 0.054 | .087* |
| 5. W1 PSQI Habitual sleep inefficiency |  |  |  | – | -.097* | 0.055 | .080* | 0.06 | 0.027 | 0.043 | 0.055 | -0.021 | 0.051 | .292* | .091* | .111* | .157* |
| 6. W1 Sleep efficiency (%) (Sleep phase) |  |  |  |  | – | -.683* | -.743* | -.433* | -.740* | -.728* | -.806* | -.547* | .383* | -.143* | -.130* | -.112* | -.120* |
| 7. W1 Total activity counts (Sleep phase) |  |  |  |  |  | – | .813* | .578* | .814* | .825* | .802* | .567* | -.312* | .151* | .192* | .110* | .149* |
| 8. W1 Mean activity counts (Sleep phase) |  |  |  |  |  |  | – | .605* | .840* | .837* | .840* | .543* | -.307* | .143* | .171* | .101* | .150* |
| 9. W1 Maximum activity counts (Sleep phase) |  |  |  |  |  |  |  | – | .562* | .562* | .532* | .382* | -.197* | .106* | .103* | .079* | .109* |
| 1. W1 WASO (Sleep phase) |  |  |  |  |  |  |  |  | – | .911* | .874* | .678* | -.400* | .118* | .170* | .120* | .141* |
| 11. W1 Wake time (Sleep phase) |  |  |  |  |  |  |  |  |  | – | .873* | .694* | -.398* | .116* | .151* | .104* | .133* |
| 12. W1 % of wake time (Sleep phase) |  |  |  |  |  |  |  |  |  |  | – | .625* | -.404* | .140* | .158* | .120* | .139* |
| 13. W1 Average wake bouts (Sleep phase) |  |  |  |  |  |  |  |  |  |  |  | – | -.505* | .070* | .131* | .113* | .100* |
| 14. W1 Average sleep bouts (Sleep phase) |  |  |  |  |  |  |  |  |  |  |  |  | – | -0.041 | -0.043 | -0.002 | -0.019 |
| 15. W1 PSQI Subjective poor sleep quality |  |  |  |  |  |  |  |  |  |  |  |  |  | – | .364* | .210* | .371* |
| 16. W1 PSQI Sleep disturbances |  |  |  |  |  |  |  |  |  |  |  |  |  |  | – | .193* | .256* |
| 17. W1 PSQI Sleep medication consumption |  |  |  |  |  |  |  |  |  |  |  |  |  |  |  | – | .144* |
| 18. W1 PSQI Daytime dysfunction |  |  |  |  |  |  |  |  |  |  |  |  |  |  |  |  | – |

*Note.* * *p* < .05.

PSQI, Pittsburgh Sleep Quality Index; W1, wave 1 (2004–2006); SOL, sleep onset latency; TST, total sleep time; WASO, wake after sleep onset.

Table S3

*Multivariate ML model performance metrics of W1 variables predicting W2 GAD severity using the GAD symptom scales that included sleep items*

| Metric | Estimate | LCI | UCI |
| --- | --- | --- | --- |
| Least absolute shrinkage and selection operator (LASSO) | | | |
| RMSE | 0.103 | 0.098 | 0.108 |
| MAE | 0.080 | 0.076 | 0.084 |
| *R*^2^ | 17.342% | 12.627% | 22.374% |
| Ridge regression | | | |
| RMSE | 0.103 | 0.098 | 0.108 |
| MAE | 0.080 | 0.076 | 0.084 |
| *R*^2^ | 16.955% | 12.232% | 21.984% |
| Elastic net regression (ENR) | | | |
| RMSE | 0.103 | 0.099 | 0.108 |
| MAE | 0.080 | 0.076 | 0.084 |
| *R*^2^ | 16.531% | 11.026% | 21.516% |
| Decision trees (DCT) | | | |
| RMSE | 0.104 | 0.099 | 0.109 |
| MAE | 0.080 | 0.076 | 0.085 |
| *R*^2^ | 15.680% | 10.680% | 20.880% |
| Random forest (RF) | | | |
| RMSE | 0.070 | 0.067 | 0.073 |
| MAE | 0.056 | 0.053 | 0.059 |
| *R*^2^ | 62.000% | 59.970% | 64.120% |
| Gradient boosting machine (GBM) | | | |
| RMSE | 0.097 | 0.093 | 0.102 |
| MAE | 0.075 | 0.071 | 0.079 |
| *R*^2^ | 26.210% | 21.560% | 31.160% |
| Support vector machine (SVM) | | | |
| RMSE | 0.109 | 0.104 | 0.115 |
| MAE | 0.078 | 0.073 | 0.083 |
| *R*^2^ | 9.520% | 6.100% | 13.570% |

*Note*. ML, machine learning; W1, wave 1 (2004–2006); W2, wave 2 (2013–2014); GAD, generalized anxiety disorder; LCI, lower bound of the 95% confidence intervals (CIs) of the bootstrapped model performance metric; UCI, upper bound of the 95% CIs of the bootstrapped model performance metric; RMSE, root mean squared error; MAE, mean absolute error; *R*^2^, R-squared.

Figure S1

*Calibration plot to assess how well predicted scores matched actual outcomes for the analyses that excluded sleep items*


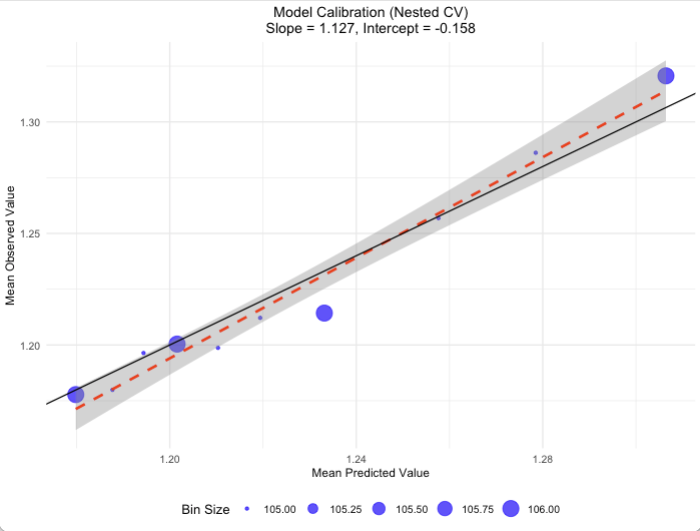


*Note.* CV, cross-validation. Each blue dot in the calibration plot above indicates a bin of predictions, such that its horizontal (*x*-axis) position denotes the average predicted value for that bin, and its vertical (*y*-axis) position indicates the matching average observed score. Further, the size of the bin signals the number of cases or observations in that bin. Calibration analysis is conducted to examine the extent to which the model’s predicted scores match the actual outcome values, reflecting how the expected values show neither systematic underestimation nor overestimation across the predictor score ranges. In the primary analysis that excluded the generalized anxiety disorder (GAD) severity sleep items at wave 1 (W1) and wave 2 (W2), the calibration slope was 1.127, suggesting a slight overestimation of predicted values. The calibration intercept was -0.153, indicating a slight global bias toward underprediction.

Figure S2

*Partial dependence plots of W1 sleep disturbance variables predicting W2 GAD severity using the GAD symptom scales that included sleep items*


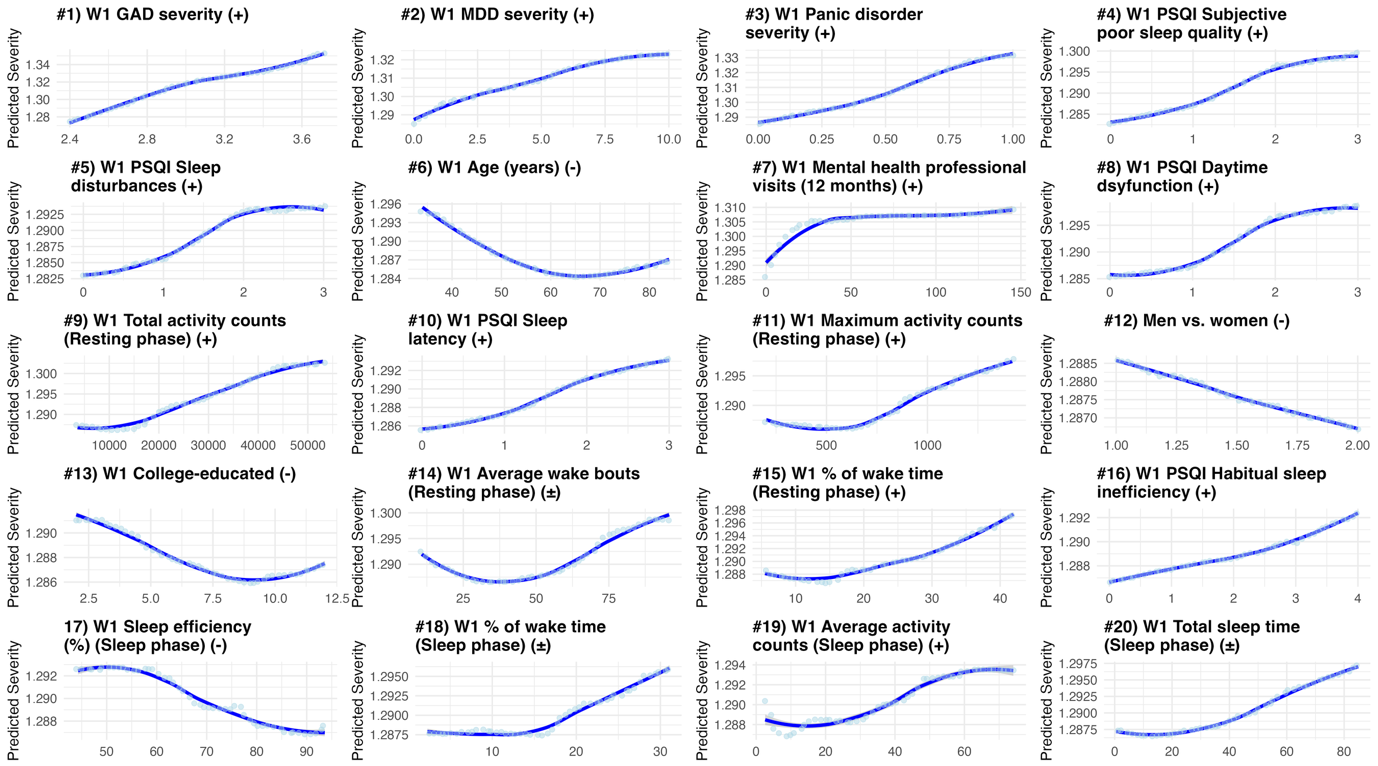


*Note*. W1, wave 1 (2004–2006); W2, wave 2 (2013–2014); GAD, generalized anxiety disorder; PD, panic disorder; MDD, major depressive disorder; SUD, substance use disorder; PSQI, Pittsburgh Sleep Quality Index.

Figure S3

*SHAP bee swarm plot of W1 sleep disturbance variables predicting W2 GAD severity using the GAD symptom scales that included sleep items*


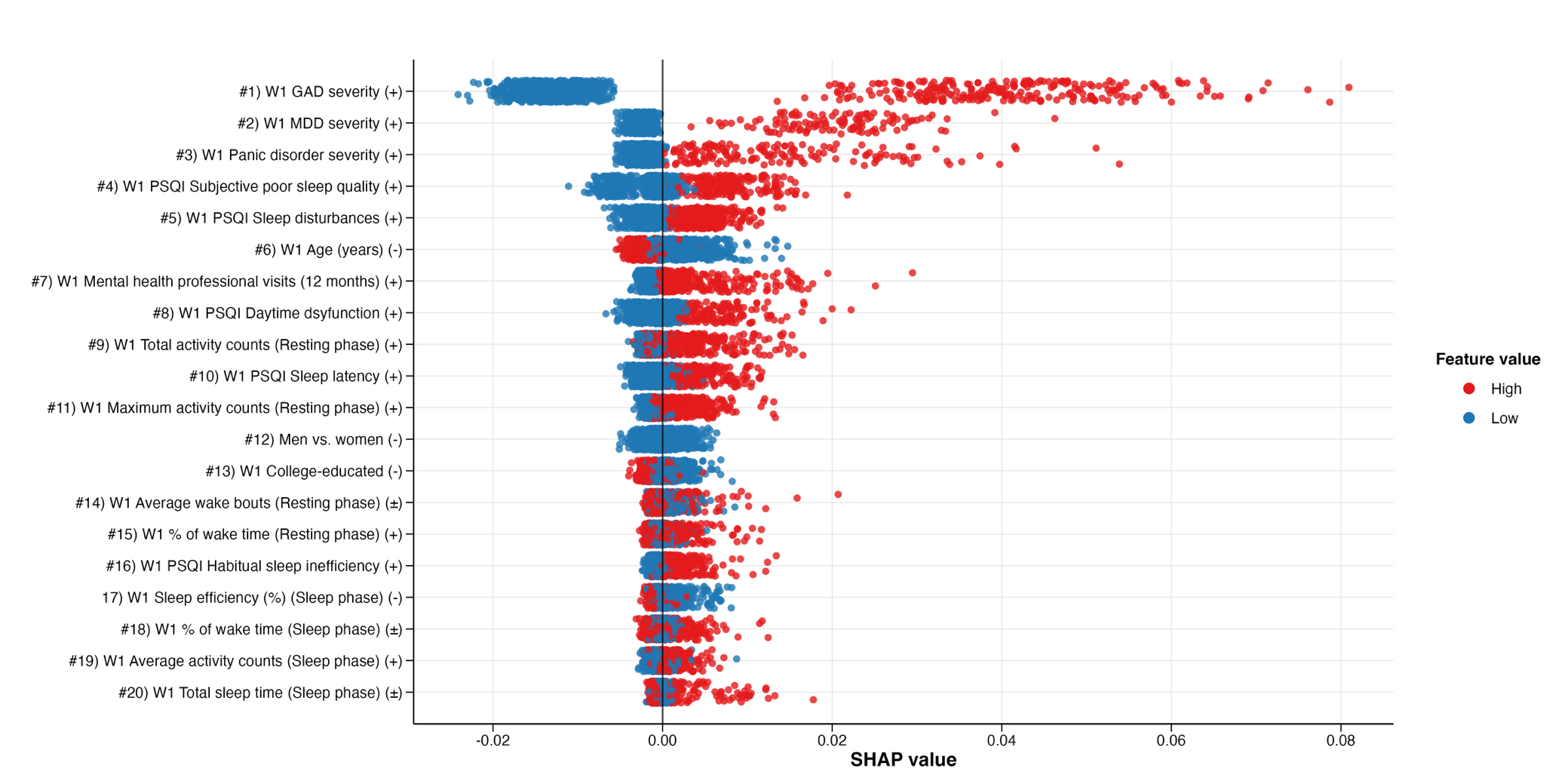


*Note*. W1, wave 1 (2004–2006); W2, wave 2 (2013–2014); GAD, generalized anxiety disorder; PD, panic disorder; MDD, major depressive disorder; SUD, substance use disorder; PSQI, Pittsburgh Sleep Quality Index.

Figure S4

*Calibration plot to assess how well predicted scores matched actual outcomes for the analyses that included sleep items*


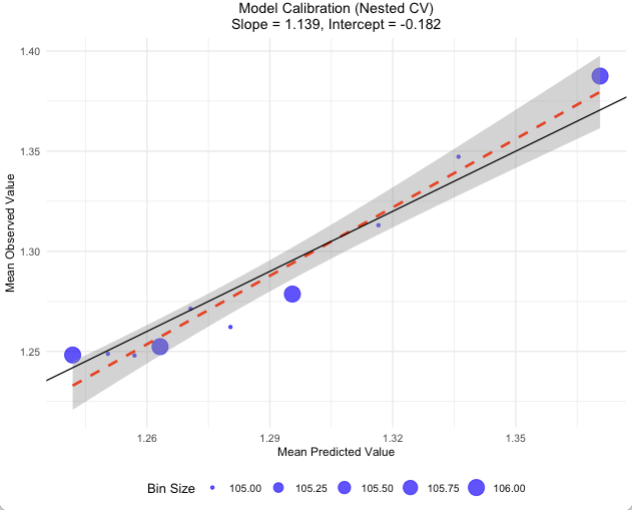


*Note.* CV, cross-validation. Each blue dot in the calibration plot above indicates a bin of predictions, such that its horizontal (*x*-axis) position denotes the average predicted value for that bin, and its vertical (*y*-axis) position indicates the matching average observed score. Further, the size of the bin signals the number of cases or observations in that bin. Calibration analysis is conducted to examine the extent to which the model’s predicted scores match the actual outcome values, reflecting how the expected values show neither systematic underestimation nor overestimation across the predictor score ranges. In the primary analysis that excluded the generalized anxiety disorder (GAD) severity sleep items at wave 1 (W1) and wave 2 (W2), the calibration slope was 1.139, suggesting a slight overestimation of predicted values. The calibration intercept was -0.182, indicating a slight global bias toward underprediction.

# References

Brand, J. E., Zhou, X., & Xie, Y. (2023). Recent developments in causal inference and machine learning. *Annual Review of Sociology*, *49*(Volume 49, 2023), 81-110. <https://doi.org/https://doi.org/10.1146/annurev-soc-030420-015345>

Buysse, D. J., Reynolds, C. F., 3rd, Monk, T. H., Berman, S. R., & Kupfer, D. J. (1989). The Pittsburgh Sleep Quality Index: a new instrument for psychiatric practice and research. *Psychiatry Research*, *28*(2), 193-213. <https://doi.org/10.1016/0165-1781(89)90047-4>

Carpi, M. (2025). The Pittsburgh Sleep Quality Index: a brief review. *Occupational Medicine*, *75*(1), 14-15. <https://doi.org/10.1093/occmed/kqae121>

David, L. K., Wang, J., & Angel, V. (2025). Validation of social science theories using machine learning models: a methodological perspective. *Quality & Quantity*, *59*(3), 2799-2823. <https://doi.org/10.1007/s11135-025-02075-0>

Dietch, J., R., Taylor, D., J., Sethi, K., Kelly, K., Bramoweth , A., D., & Roane, B. M. (2016). Psychometric evaluation of the PSQI in U.S. college students. *Journal of Clinical Sleep Medicine*, *12*(08), 1121-1129. <https://doi.org/10.5664/jcsm.6050>

Donahue, C. C., Smulligan, K. L., Wingerson, M. J., Brna, M. L., Simon, S. L., Wilson, J. C., & Howell, D. R. (2025). Clinical cut point for the Pittsburgh Sleep Quality Index after adolescent concussion. *Orthopaedic Journal of Sports Medicine*, *13*(4), 23259671251330571. <https://doi.org/10.1177/23259671251330571>

Kovač, N., Ratković, K., Watson, P., Farahani, H., & Bagheri Sheykhangafshe, F. (in press). Machine learning classification models for predicting chronic pain. *Current Psychology*. <https://doi.org/10.1007/s12144-025-08294-w>

Kyriazos, T., & Poga, M. (2024). Application of machine learning models in social sciences: Managing nonlinear relationships. *Encyclopedia*, *4*(4), 1790-1805. <https://doi.org/10.3390/encyclopedia4040118>

Lavelle-Hill, R., Smith, G., Deininger, H., & Murayama, K. (in press). An explainable artificial intelligence handbook for psychologists: Methods, opportunities, and challenges. *Psychological Methods*. <https://doi.org/10.1037/met0000772>

Lavelle-Hill, R., Smith, G., & Murayama, K. (2025). Bridging traditional-statistics and machine-learning approaches in psychology: Navigating small samples, measurement error, nonindependent observations, and missing data. *Advances in Methods and Practices in Psychological Science*, *8*(3), 25152459251345696. <https://doi.org/10.1177/25152459251345696>

Li, X., Jacobucci, R., & Ammerman, B. A. (2021). Tutorial on the use of the regsem package in R. *Psych*, *3*(4), 579-592. <https://doi.org/10.3390/psych3040038>

Shahid, A., Wilkinson, K., Marcu, S., & Shapiro, C. M. (2011). Pittsburgh Sleep Quality Index (PSQI). In A. Shahid, K. Wilkinson, S. Marcu, & C. M. Shapiro (Eds.), *STOP, THAT and One Hundred Other Sleep Scales* (pp. 279-283). Springer. <https://doi.org/10.1007/978-1-4419-9893-4_67>
